# Cyclodextrin-Grafted Polysaccharides as Sustainable Platforms for Biomedical and Environmental Applications

**DOI:** 10.3390/ijms27156566

**Published:** 2026-07-23

**Authors:** Elisabetta Lacolla, Clarissa Ciarlantini, Iolanda Francolini, Eleftheria Dossi, Antonella Piozzi

**Affiliations:** 1 Chemistry Department, Sapienza University of Rome, Piazzale Aldo Moro 5, 00185 Rome, Italy; elisabetta.lacolla@uniroma1.it (E.L.); clarissa.ciarlantini@uniroma1.it (C.C.); iolanda.francolini@uniroma1.it (I.F.); 2 Centre for Defence Engineering and Physical Science, Cranfield University, Defence Academy of UK, Shrivenham SN6 8LA, UK; e.dossi@cranfield.ac.uk

**Keywords:** cyclodextrins, polysaccharides, functionalization, grafting, drug delivery, pollutant removal

## Abstract

Cyclodextrins (CDs) offer a promising solution for enhancing the aqueous solubility of various hydrophobic molecules and facilitate their transport and delivery. Although CDs possess inherent molecular recognition capabilities, their derivatization is often essential to augment solubility and functional performance. A promising strategy involves the grafting of cyclodextrins onto polysaccharides. These hybrid systems combine the molecular recognition of CDs with the structural stability and versatility of biopolymers, yielding advanced materials suitable for biomedical and environmental applications. This review provides a comprehensive overview of CDs, focusing on their unique structural characteristics and their ability to form host-guest inclusion complexes with various hydrophobic molecules. The diverse methodologies used for CD modification and their immobilization onto polysaccharide backbones are described. Furthermore, the multifaceted applications of these grafted materials are explored. While in the pharmaceutical field these systems serve as advanced drug delivery platforms to improve the bioavailability and stability of hydrophobic therapeutic agents, in the environmental sector they demonstrate exceptional efficiency in wastewater treatment, acting as potent sorbents for the removal of organic pollutants and heavy metals. By bridging fundamental chemistry with practical applications, this review highlights the potential of CD-polysaccharide hybrids as sustainable solutions to pressing clinical and ecological challenges.

## 1. Introduction

The inherent low solubility in aqueous media of hydrophobic molecules poses significant challenges for their use across various applications, including pharmaceuticals and environmental science [1,2]. The pronounced hydrophobicity of many therapeutic agents and active ingredients—including those of natural origin—severely limits their clinical utility, as poor absorption and low bioavailability impair their therapeutic efficacy [3]. From an environmental standpoint, hydrophobic organic molecules such as pesticides, polycyclic aromatic hydrocarbons (PAHs), and persistent lipophilic compounds represent a majority portion of pollutants in groundwater and soil [4]. These compounds are discharged into water resources through industrial and urban effluents, accumulating in living organisms with deleterious effects. Given the increasing urgency of these issues, recent scientific research has focused on developing innovative systems to mitigate such concerns [5]. Of particular interest is the development of versatile platforms capable of providing controlled release of bioactive molecules and remediating pollutants. In this context, biopolymers are emerging as materials with great potential due to their natural origin, biocompatibility, biodegradability, and cost-effectiveness [6,7]. Specifically, polysaccharides such as chitosan, alginate, dextran, cellulose, starch, hyaluronic acid, and pectin occupy a prominent position due to their eco-sustainability and amenability to chemical modification, providing highly versatile platforms for a broad spectrum of applications [8,9,10,11]. Complementing these polysaccharide systems, cyclic oligosaccharides such as cyclodextrins (CDs)—derived from the enzymatic degradation of starch—offer unique advantages for the encapsulation and transport of hydrophobic molecules. CDs possess a distinctive truncated cone structure, characterized by a central hydrophobic cavity and a hydrophilic outer surface. This architecture enables the formation of host-guest inclusion complexes with various hydrophobic moieties, sequestering them within the internal cavity and thereby enhancing their solubility in aqueous environments [12,13]. Despite these favorable properties and their inherent biocompatibility, the use of native CDs is often hampered by the relative instability of their inclusion complexes, primarily due to weak non-covalent interactions [14]. Such instability can lead to premature or uncontrolled release of the guest molecule. Furthermore, the intrinsic aqueous solubility of natural CDs, particularly βCD, is relatively low, further constraining their applicability in specific routes of administration when applied for drug delivery.

To circumvent these limitations, advanced CD-based architectures have been engineered, such as those where CDs are crosslinked into a network or grafted onto polymeric backbones [15,16,17,18,19,20,21]. This strategy not only enhances their structural stability and mechanical integrity but also significantly broadens the functional scope of CD-based materials. The present review provides a critical and comprehensive evaluation of sustainable, biocompatible polysaccharide systems incorporating functionalized CDs for the encapsulation of hydrophobic guests. The impact of CD chemical modifications offers a diversity of CD–polysaccharide interactions—ranging from covalent bonding to supramolecular assembly—and enhancement of encapsulation efficiency and release kinetics. Furthermore, this work elucidates the relationship between the structural features of these materials and their performance in specific applications, highlighting their dual potential in targeted drug delivery and environmental remediation. Finally, the prevailing limitations, persistent challenges, and the future outlook for the development of multifunctional platforms based on the integration of polysaccharides and functionalized CDs are critically discussed. This review covers research studies published between 2016 and 2026, offering a comprehensive overview of the current state of the art and emerging trends in the field.

## 2. Cyclodextrins: Structure and Properties

**Figure 1:**
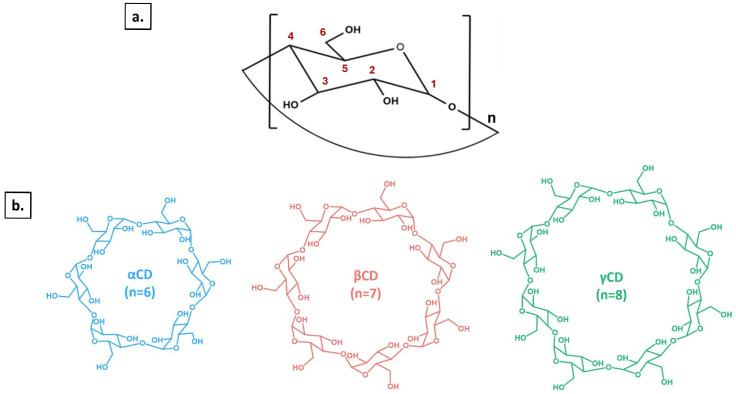
Schematic representation of (**a**) α-D-glucopyranoside, the repeating unit of CDs, and (**b**) α-, β-, and γ- cyclodextrins.

Cyclodextrins are cyclic oligosaccharides produced through the enzymatic degradation of starch by cyclodextrin glycosyltransferase (CGTase) [22]. The three primary natural Cyclodextrins—α-, β-, and γ-CD (Figure 1)—are distinguished by their number of glucose units (6, 7, and 8, respectively). They units are linked by α-1,4-glycosidic bonds and adopt a toroidal conformation characterized by a hydrophilic exterior surface and a hydrophobic internal cavity.

This unique structure enables CDs to form host-guest inclusion complexes with lipophilic moieties, thereby enhancing their solubility and stability in aqueous media [23,24]. Consequently, their application has become widespread across diverse scientific and industrial sectors, driving innovation in drug delivery, environmental remediation, and materials science. X-ray crystallography studies have established that the primary hydroxyl groups (at C6) are clustered at the narrower rim of the toroidal structure, whereas the secondary hydroxyl groups (C2 and C3) are situated on the wider face (Figure 1) [25]. The cavity’s interior is lined by non-polar hydrogen atoms (bonded to C3 and C5) and ether-like oxygen atoms. Notably, while the primary hydroxyl groups exhibit rotational freedom—effectively narrowing the cavity aperture—the secondary hydroxyl groups participate in a hydrogen-bonding network that imparts structural rigidity [26]. The structural and physicochemical properties of α-, β-, and γ-CD are summarized in Table 1.

As indicated in Table 1, the aqueous solubility of native CDs is generally limited. Notably, β-CD exhibits significantly lower aqueous solubility than its counterparts. This phenomenon is attributed to a circumferential network of intramolecular hydrogen bonds between the C2-OH and C3-OH groups of adjacent glucose units. The inherent low solubility of native CDs often necessitates chemical functionalization to improve their solubility, stability, and selectivity, thereby broadening their industrial and pharmaceutical utility [27].

**Table 1:** Structural and physicochemical properties of α-, β-, and γ-cyclodextrins.

Cyclodextrin	α	β	γ
Number of glucose units	6	7	8
Molar mass (g/mol)	972	1135	1297
Solubility in water at room temperature (g/L) [28]	0.145	0.0185	0.232
Cavity diameter (Å) [29]	4.7–5.3	6–6.5	7.5–8.3
[α]D 25 °C [22]	150 ± 5	162 ± 0.5	177.4 ± 5
Height of torus (Å) [30]	7.9 ± 0.1	7.9 ± 0.1	7.9 ± 0.1
Outer diameter (Å) [30]	14.6 ± 0.4	15.4 ± 0.4	17.5 ± 0.4
Cavity volume (Å) [30]	174	262	427
Approx. cavity volume in 1 mol CD (mL) [22]	104	157	256
Crystal forms (from water) [31]	Hexagonal plates [32,33]	Monoclinic parallelograms [33]	Quadratic prisms [33]
Crystal water (% *w*/*w*) [22,31,34]	10.2	13.2–16.0	8.13–20.3
Diffusion constant at 40 °C [22]	3.443	3.224	3.000
pK (by potentiometry) at 25 °C [22]	12.332	12.202	12.081
Surface Tension (mN/m) [12]	71	71	71

Natural CDs exist as hydrates, incorporating water molecules both within their cavities and through their crystal lattices. The water content and the specific dehydration temperatures vary among CDs, reflecting the relative binding affinity of the sequestered water. As shown in Table 2, α- and β-CD undergo mass loss due to dehydration within a similar temperature range. In contrast, γ-CD, which possesses the highest water solubility, releases water at lower temperatures, indicating that the water molecules are less stringently retained within its framework. CDs may also undergo phase transitions, particularly in their anhydrous states or during the dehydration process [35,36]. These transitions are primarily driven by solid-state structural rearrangements resulting from the loss of crystalline water, which lead to distinct anhydrous polymorphic forms before thermal degradation. Table 2 summarizes the thermal parameters obtained via thermogravimetric analysis (TGA) and differential scanning calorimetry (DSC). Regarding thermal decomposition, α-CD shows an onset around 270 °C, whereas β-CD degradation begins at 250 °C, with ignition occurring above 300 °C in air. γ-CD exhibits the greatest thermal stability, with decomposition observed in the range 293–324 °C [37,38].

## 3. Derivatization of Cyclodextrins

**Figure 2:**
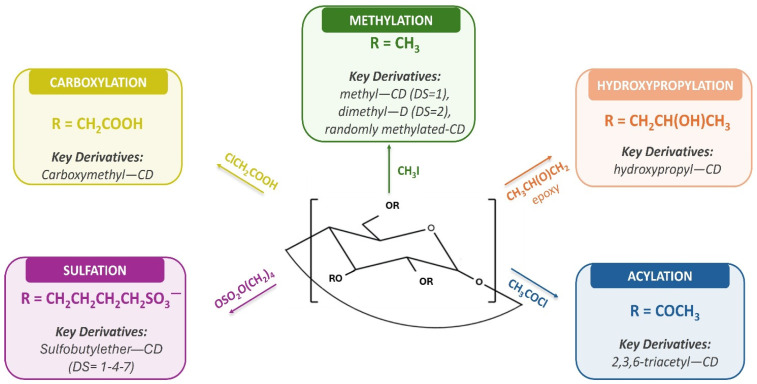
Examples of functionalized CDs.

Among the physicochemical properties of native compounds, the low aqueous solubility of β-CD represents a significant limitation. Furthermore, their efficacy can be hindered by the formation of complexes with insufficient thermodynamic stability. To overcome these limitations, CDs are often functionalized by introducing specific substituents; their abundance of reactive hydroxyl groups renders these oligosaccharides particularly suitable for chemical modification. Through the strategic derivatization of primary and secondary hydroxyl groups, CD derivatives can be tailored to meet specific requirements, thereby significantly enhancing their solubility, binding affinity, and molecular selectivity [39]. The hydroxyl groups at positions C-2, C-3, and C-6 often compete for reagents, frequently complicating selective modification. Generally, the primary hydroxyl groups on the C-6 carbons exhibit higher reactivity due to their greater spatial accessibility and reduced steric hindrance, rendering them more susceptible to derivatization. From a chemical perspective, the C-6 hydroxyl groups are the most basic and typically the most nucleophilic; conversely, the C-2 hydroxyl groups are the most acidic, while those at the C-3 position are the most sterically hindered [26]. Under standard conditions, the C-6 position is the most prone to electrophilic attack. Consequently, the use of lower-reactivity reagents, such as butylmethylsilyl chloride (TBDMSCl), facilitates greater regioselectivity toward these primary hydroxyl groups. In contrast, highly reactive reagents, such as trimethylsilyl chloride (TMSCl), tend to react indiscriminately with both primary and secondary hydroxyl groups [40]. Among the native forms, β-CD is the most frequently modified CD due to several factors, including its lower production costs, its characteristically low aqueous solubility, and a broader range of applications compared to other CD types. The most common chemical modifications of CDs are outlined below and summarized in Table 3:

**Methylation:** Methylated Cyclodextrins (Figure 2) are typically synthesized by treating native CDs with methyl iodide to yield derivatives such as methyl-β-cyclodextrin (M-β-CD). This modification increases the hydrophobicity of the CD cavity while simultaneously improving its solubility in both water and organic solvents, thereby enhancing its capacity to solubilize hydrophobic guest molecules [41,42]. For instance, dimethyl-β-cyclodextrin (DM-β-CD) is characterized by the substitution of two hydroxyl groups on each glucopyranose unit. DM-β-CD is noted for its improved safety profile compared to native β-CD, rendering it highly suitable for drug delivery and the encapsulation of lipophilic compounds [43]. Superior water solubility can be achieved by methylating β-CD to obtain heptakis(2,3,6-tri-O-methyl)-β-CD (TRIMEB), which exhibits high aqueous solubility alongside a hydrophobic cavity. This modification increases the system’s affinity for antibacterial agents and highly hydrophobic molecules, such as cholesterol. For instance, while inclusion complexes with pipemidic acid enhance the bioavailability of the antibacterial agent—thereby reducing both the required dose and side effects—the formation of TRIMEB-cholesterol complexes via cholesterol extraction from cell membranes increases the permeability of biological barriers, thus promoting drug absorption [44,45]. Randomly methylated β-CD (RM-β-CD) is produced through stochastic methylation, resulting in a complex mixture of various substitution patterns. This randomness provides an optimal balance between hydrophilicity and hydrophobicity, making RM-β-CD highly versatile for various applications, including pharmaceutical formulations, the food industry, and analytical chemistry, particularly in chiral separation techniques such as capillary electrophoresis [46,47,48]. The resulting structural variability leads to unique interaction profiles with guest molecules, enabling tailored complexation properties [49].

**Hydroxypropylation:** This derivatization involves the introduction of hydroxypropyl groups into the CD structure (Figure 2). This modification not only significantly enhances aqueous solubility but also improves the capacity to form inclusion complexes with a wide range of drugs, thereby facilitating controlled release and targeted drug delivery [50]. Studies have demonstrated that hydroxypropylation at the O-2 position results in a more extended configuration, whereas substitution at the O-6 position reduces the water density within the cavity [51]. Due to this functionalization, the solubility of hydroxypropyl-β-cyclodextrin (HP-β-CD) can exceed 600 g/L; this drastically reduces the crystallinity of the native CD and enhances its overall complexation efficiency [52]. As one of the most critical derivatives, HP-β-CD is synthesized through the reaction of β-CD with propylene oxide. It is currently considered the most versatile excipient among cyclic oligosaccharides. Its widespread pharmaceutical application is supported by its regulatory approval for multiple routes of administration, including parenteral, oral, ocular, topical, and rectal [53,54]. HP-β-CD is successfully employed in the solubilization of poorly water-soluble active pharmaceutical ingredients, such as dexamethasone and itraconazole (ITZ) [55].

**Acylation**: Modifying CDs through the introduction of acyl groups (Figure 2) allows for the precise modulation of both solubility and lipophilicity. These acylated variants are engineered for specific applications in drug delivery systems, ensuring enhanced stabilization of the active ingredient and optimized release profiles. Acylation primarily aims to increase lipophilicity and improve solubility in organic solvents. The functionalization typically occurs via the esterification of hydroxyl groups using acetic anhydride or acetyl chloride. In particular, peracetylation (the replacement of all hydroxyl groups) is readily achieved using an excess of acetic anhydride under either acid or base catalysis [26,46,56,57].

**Table 3:** Overview of CD derivatives, including modifications, degree of substitution, advantages, applications and references.

Modification	Degree of Substitution (DS)	Advantages	Application	Reference
Methylation				
Dimethyl-β-cyclodextrin (DM-β-CD)	2 OH groups substituted per glucopyranose unit	Better safety profile than native β-CD	Drug delivery and encapsulation of lipophilic compounds	Giri et al. [43]
Randomly methylated β-cyclodextrin (RM-β-CD)	Variable/random	Optimal balance between hydrophilicity and hydrophobicity	Pharmaceutical formulations, food industry, analytical chemistry	Musuc et al. [46]; Spiridon et al. [47]; De Gaetano et al. [48]; Ding et al. [49]
Total methyl β-cyclodextrin (TRIMEB)	3 OH groups substituted per unit	Highly lipophilic, crystalline derivative; enhanced aqueous solubility; high affinity for highly hydrophobic molecules; increases the permeability of biological barriers	Promotion of drug absorption via cholesterol inclusion complexes	Nishijo et al. [44]; Lavorgna et al. [45]
Hydroxypropylation				
Hydroxypropyl-β-cyclodextrin (HP-β-CD)	~0.4–1.5 substituents per glucopyranose unit	High water solubility; drastic reduction in crystallinity; increased complexation efficiency and biocompatibility	Widely used pharmaceutical excipient approved for multiple administration routes (parenteral, oral, ocular, topical, rectal)	Li et al. [50]; Yong et al. [51]; Sandilya et al. [52]; Suta et al. [55]; Malanga et al. [54]; Paul et al. [53]
Acylation:				
2,3,6-triacetyl-β-cyclodextrin (TA-β-CD)	3 OH groups substituted per unit	Increases lipophilicity; high solubility in organic solvents; extends biological half-life, and sustains release of hydrophilic drugs via water-insoluble drug/CD complexes	Sustained-release carrier for hydrophilic drugs	Kuplennik et al. [56]; Yuan et al. [57]
Sulfation				
Sulfobutylether-β-cyclodextrin (SBE-β-CD)	DS 1, 4, and 7 are most widely used in pharmaceutical formulations (0.14–1 substituents per glucopyranose unit)	Negatively charged sulfonate groups significantly improve aqueous solubility; electrostatic interaction capabilities; high biocompatibility, increasing drug efficacy/potency while reducing therapeutic dose and toxicity	Injectable pharmaceutical products (FDA-approved),	Stella et al. [58]; Musuc et al. [46]; Eid et al. [59]
Carboxylation				
Carboxymethyl-β-cyclodextrin (CM-β-CD)	Variable DS (low to high) up to crosslinked networks	Improves water solubility of native CDs; reduces toxicity	Controlled/targeted drug delivery systems (e.g., pH-sensitive carriers), exploiting improved solubility and stimuli-responsive complexation behavior	Yang et al. [60]; Allahyari et al. [61]; Kali et al. [62]; Hadadian et al. [63]

**Sulfation and phosphorylation**: These modifications introduce charged groups onto the CD framework (Figure 2), increasing hydrophilicity and enhancing interactions with biological systems. Customization through the attachment of various functional groups (e.g., amino, carboxyl, or thiol groups) tailors the interactive properties of CDs, improving their performance in chiral applications, drug delivery, and targeted therapies. Among these derivatives, sulfobutylether-β-cyclodextrin (SBE-β-CD) is the most prominent. Its negatively charged sulfonate groups significantly improve aqueous solubility and provide electrostatic interaction capabilities, especially with cationic drugs [46,58,59].

**Carboxylation**: The fundamental reaction involved is esterification (Figure 2), occurring with anhydrides or polycarboxylic acids such as citric acid, polyacrylic acid, or pyromellitic dianhydride. Depending on the reaction conditions, it is possible to obtain either water-soluble functionalized CDs or insoluble cross-linked CD polymers. Carboxymethyl-β-cyclodextrin (CM-β-CD) is the most widely used derivative, synthesized typically in a basic environment using chloroacetic acid or its sodium salt [60]. Such modifications significantly improve the water solubility of native CDs, reduce toxicity, and enhance the formation of inclusion complexes with hydrophobic or cationic drugs [61,62,63,64,65].

Therefore, the selection of a specific derivative is strictly dictated by its intended application. Furthermore, the degree of substitution (DS) is pivotal in modulating the behavior of these macrocycles. The DS directly governs critical parameters such as aqueous solubility, structural stability, and complexation efficiency, ultimately determining the performance of the CD-based system in its target environment. Table 3 summarizes various CD derivatives, including their modifications, DS, advantages, applications and relevant references.

Beyond simple chemical derivatives, current research increasingly focuses on engineering advanced CD-based architectures. These include crosslinked CD networks [66], CDs grafted onto polymeric backbones, and interlocked supramolecular structures, such as polyrotaxanes and pseudorotaxanes, which significantly improve the physicochemical properties and functional versatility of these oligosaccharides [26,67,68]. Due to their inherent biocompatibility and structural adaptability, CDs remain a cornerstone of supramolecular chemistry, with expanding potential for high-impact applications.

## 4. Inclusion Complexes (ICs)

**Figure 3:**
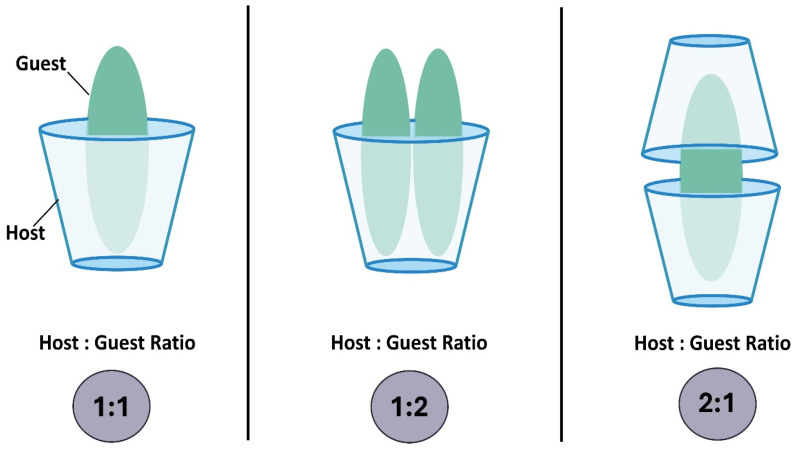
Possible stoichiometry of host–guest complexes.

As mentioned previously, the primary characteristic of CDs is their ability to form inclusion complexes (ICs) with hydrophobic molecules through noncovalent interactions, subsequently allowing for the controlled release of the guest. The driving forces governing complexation include hydrophobic, electrostatic, and dipole–dipole interactions, as well as van der Waals forces, hydrogen bonds, and charge-transfer interactions [30]. To maximize favorable interactions and improve IC stability, guest molecules spontaneously adopt a preferred orientation that optimizes contact between their hydrophobic moieties and the nonpolar host CD cavity [30,69]. These host-guest complexes alter the physicochemical properties of the encapsulated molecules, resulting in increased solubility, bioavailability, and chemical stability. Furthermore, they reduce volatility and toxicity, mitigate unwanted side effects, and mask unpleasant odors or flavors [70]. Beyond pharmaceuticals, their hydrophobic cavity enables the selective removal of organic pollutants from aqueous matrices [71]. When a guest molecule enters the CD cavity, it displaces highly ordered, high-energy water molecules, leading to the formation of a more stable host-guest complex [34,72]. This process is typically driven by enthalpic contributions. Entropic contributions arise primarily from the increased disorder of the water molecules once they are released from the restricted cavity [39,73]. The stability of the resulting complex is quantified by the formation (association) constant *K_f_*, where higher values indicate a more robust complex [74]. Encapsulation generally enhances water solubility, an effect evaluated using the Higuchi-Connors (1965) phase solubility method. This method analyzes the variation in guest solubility as a function of CD concentration. A linear profile usually indicates a 1:1 stoichiometry and allows for the calculation of the apparent stability constant (*K*_s_) and complexation efficiency (CE) [74]. Deviations from linearity may indicate complex instability or higher-order stoichiometries [75]. The stoichiometry of these complexes depends on both thermodynamic and steric factors. While the 1:1 ratio is the most common, 2:1 (two drugs in one CD) or 1:2 (one drug spanning two CDs) ratios can also form (Figure 3), depending on the size of the guest [76,77]. This ratio is experimentally determined using the Job plot (method of continuous variation) [78,79]. ICs can be prepared using solid-state, semisolid-state, or solution-based methods [80], including physical mixing [81], kneading [82], co-precipitation [83], solvent evaporation [84], spray drying, microwave irradiation [85], and supercritical fluid technology [86].

## 5. Design Strategies for Cyclodextrin-Based Systems

Although the chemical functionalization of CDs enhances their intrinsic properties and expands their range of applications, current research has shifted beyond the modification of individual molecules. These oligosaccharides become even more fascinating when integrated into complex structural systems. The use of CDs as monomeric (isolated) units presents significant limitations, primarily regarding loading efficiency. In fact, solid drug/CD complexes often contain less than 5–10% of the active ingredient. Consequently, a substantial amount of CD is required to complex even small quantities of a drug. This excess can negatively impact formulation volume, increase production costs, and potentially lead to toxicological concerns [64]. To address these critical issues, scientific literature highlights the advantages of multivalent CD-based systems. It is reported that when CD units are organized in proximity within complex architectures, they can act cooperatively, shifting the equilibrium toward complex formation and significantly enhancing the stability of the inclusion complex. Furthermore, within more complex systems, key characteristics such as solubility and toxicity can be tailored, thereby expanding their potential applications. These features make CDs particularly attractive as building blocks for advanced supramolecular architectures [87,88].

**Figure 4:**
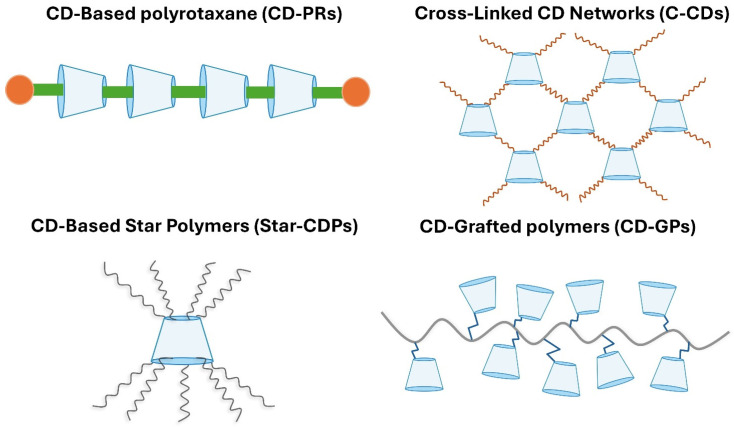
Classification of CD-polymers based on their structure.

Various classifications exist for CD-based polymer systems. In a recent and comprehensive review, Liu et al., categorized these polymers into four distinct classes according to their structures: CD-based polyrotaxanes (CD-PRs), CD-grafted polymers (CD-GPs), cross-linked CD networks (C-CDs), and CD-based star polymers (star-CDPs) (Figure 4) [89]. To complete this classification, Table 4 summarizes the architectural designs, advantages, applications, and representative case studies of cyclodextrin-based systems.

**CD-based PolyRotaxanes (CD-PRs)**: A rotaxane is a topological supramolecule consisting of a ring-shaped molecule, such as a CD, threaded onto a linear polymer axis and stabilized by bulky “stoppers” at the ends to prevent dethreading [67]. When multiple rings are threaded onto a single polymer chain, a polyrotaxane (PR) is obtained, in which the sliding-ring structure is maintained through mechanical interlocking. Similar structures include pseudopolyrotaxanes (PPRs), where CDs interact with the polymer axis via non-covalent host-guest interactions but lack bulky end-capping groups [90]. CD-based PRs have recently attracted significant attention owing to their unique biocompatibility and structural versatility. Due to the excellent complexation properties of CDs, these host-guest interactions easily lead to the formation of these interlocked, necklace-like supramolecular architectures. Moreover, the ability of CDs to rotate and slide along the polymer axis, combined with the possibility of chemical modification via their numerous –OH groups, plays a crucial role. One of the most widely used CD-PRs is formed by CDs as the host and poly(ethylene glycol) (PEG) as the guest polymer [91]. Regarding applications, α-CD/PEG polyrotaxanes have been extensively explored as biomaterials and drug delivery systems [92]. Their unique architecture, characterized by the sliding motion of the α-CDs along the PEG chain, contributes significantly to their functional properties. These systems can be used to develop hydrogels for sustained drug release, intracellular drug delivery, and targeted drug delivery through specific ligand functionalization. Recently, Cardoso et al. created supramolecular gels based on poly(pseudo)rotaxane-cyclodextrin architectures using supercritical CO_2_ for the transdermal delivery of carvedilol [93]. The α-CD-containing systems showed increased in vitro diffusion and increased ex vivo skin permeability, suggesting potential interactions between CD and the skin membrane. Furthermore, Taharabaru et al. developed CD-based aminated polyrotaxanes, in which multiple α-CDs strung on a PEG shaft form dynamic supramolecular carriers capable of efficiently delivering the Cas9 ribonucleoprotein, with optimized structures that significantly improve endosomal escape and the efficacy of genome editing [94].

**Cross-linked Cyclodextrins (C-CDs)**: C-CDs are obtained from the reaction between the hydroxyl groups of CDs and crosslinking agents such as aldehydes, ketones, isocyanates, citric acid and epoxides (e.g., epichlorohydrin and glycidyl ethers). The condensation reaction typically requires the use of chemical catalysts—such as strong acids or bases—and careful temperature control. In C-CDs, cyclodextrins are the main component, and their empty cavities can subsequently be used to encapsulate guest molecules. However, the mobility of the CDs in these systems is limited due to the highly crosslinked structure [89]. The wide range of available crosslinking molecules and different strategies enable the synthesis of materials with highly tunable properties. This large family of CD-based crosslinked porous structures comprises several types of systems, including nanosponges and metal–organic frameworks (CD-MOFs), which are of particular interest [97]. In CD-MOFs, CDs act as ligands, linking metal ions or clusters to form a three-dimensional porous structure. Taking advantage of this structure, Jia et al. fabricated a series of β-CD-derived hydrogels and reinforced this porous structure through the incorporation of a polymer network. These gels showed affinities for Au^3+^, Ag^+^, and Pb^2+^ simultaneously, with maximum capacities of 316.4, 60.9, and 414.2 mg/g, highlighting their promising potential for the treatment of wastewater containing various heavy metal ions [96]. These materials are known for their high porosity, robustness, and biocompatibility. Consequently, they find applications in areas such as gas adsorption (particularly CO_2_) and sensing, the separation of isomers and enantiomers, and controlled drug delivery [95]. The term nanosponge (NS) refers to a class of insoluble materials characterized by nanoscale porosity and significant adsorption and complexation properties. The final properties of cyclodextrin-based nanosponges (CD-NS) depend heavily on the nature of the crosslinking agent and the degree of crosslinking. These systems offer significant advantages over native CDs, such as the ability to form complexes with a wider range of guest molecules. This is due to the presence of interstitial spaces between the CD units, which can also accommodate hydrophilic compounds. Furthermore, the polymer network surrounding the CD cavities hinders the diffusion of guest molecules, allowing for slower and more controlled release [98]. Finally, the insolubility of nanosponges facilitates their easy recovery and recycling from aqueous media, making them high-performance systems for the capture of environmental pollutants [102]. There are also examples of C-CDs in which the crosslinker is a low molecular weight polymer chain, such as poly(ethylene glycol) diglycidyl ether (PEGDGE). Specifically, β- and γ-crosslinked CDs preserve the characteristics of the crosslinked network while retaining their solubility in water and alcohols [99,100]. Due to this expanded hydrodynamic volume, these systems are capable of entrapping hydrophobic molecules within their supramolecular structure, thereby significantly enhancing the encapsulation efficiency of the CDs [101].

**CD-based star polymers (star-CDPs)**: In these systems, the CD represents the central core from which linear polymer chains radiate. Specifically, thanks to the presence of hydroxyl groups, the CD can act as an initiation site for polymerization. These systems can be synthesized through various methods, typically via controlled/living radical polymerization techniques such as Reversible Addition-Fragmentation chain-Transfer (RAFT), Ring-Opening Polymerization (ROP), and Atom Transfer Radical Polymerization (ATRP) [103]. In these systems, the CD cavity remains unobstructed, allowing it to accommodate guest molecules even after the formation of the polymeric arms. Moreover, the grafted polymeric arms can be engineered to respond to various stimuli. For example, arms sensitive to factors such as pH, reactive oxygen species (ROS), temperature, or redox-active substances can be introduced.

These systems are particularly promising for biomedical applications, including targeted drug delivery, gene delivery, and diagnostic imaging [104]. However, due to their core–shell architecture, these CD-based star polymers can also be used in environmental remediation. For example, Kiran et al. developed a β-CD-centered star copolymer that acts as an efficient flocculant for wastewater treatment, improving turbidity removal through enhanced polymer-particle interactions [105].

**CD-grafted polymers (CD-GPs)**: In this architecture, CDs are grafted onto a polymer backbone or a substrate, which then serves as a polymeric host for various guests. CD-GPs are CD-dense systems where the internal cavities remain unoccupied and ready to accommodate guest molecules. Consequently, these systems can directly form inclusion complexes with drugs and other bioactive compounds. CDs can be grafted onto a variety of linear, branched, cationic, anionic, and block copolymers [90]. By combining the complexation capabilities of CDs with the structural advantages of polymers—such as mechanical strength, processability, and versatility—these hybrid materials achieve superior performance [106]. Their synthesis typically involves modifying CDs by replacing hydroxyl groups with functional groups like thiols, amines, azides, or carboxyls to facilitate attachment to the polymer chain [90]. The potential architectures of this type are numerous, offering a wide range of applications. For instance, grafting CDs onto synthetic polymers like acrylates (e.g., Poly-N-isopropylacrylamide, PNIPAM [107], Poly(N-(2-hydroxyethyl)acrylamide), PHEAm [108], and polymethylacrylate, PMA [109]) can yield noteworthy stimuli-responsive systems, particularly for medical applications [90]. Celebioglu et al. reported HP-β-CD-based poly-CD fibers functionalized with poly(acrylic acid), which improved heavy-metal removal efficiency by up to 49% compared to pristine fibers, reaching 80–100% removal at low metal concentrations [110]. Even more promising are systems where CDs are grafted onto natural polysaccharides. These hybrids enhance biocompatibility and functionality while ensuring biodegradability and non-toxicity. Furthermore, their versatility and relatively low cost make them highly attractive hybrid systems, which will be discussed in further detail below. In the following section, particular emphasis will be placed on systems based on CD–polysaccharide combinations.

## 6. Cyclodextrins Grafted onto Polysaccharides

As previously reported, the family of CD-grafted polymers includes systems in which CDs are chemically linked to polysaccharide chains. These natural polymers, composed of monosaccharide units linked by glycosidic bonds, can be extracted from a wide range of sources, including plants, microorganisms, and animals. They are particularly attractive building blocks due to their biocompatibility, biodegradability, low cost, and robust mechanical properties [111]. Their versatility stems from the presence of reactive functional groups—such as hydroxyl, carboxyl, and amino groups—which allow for facile chemical modification. The functionalization of polysaccharides with CDs is one of the most promising strategies for developing high-performance materials. In these systems, the intrinsic structural properties of the polysaccharides are combined with the inclusion capacity, molecular recognition, and controlled release profile of the CDs [112]. Consequently, advanced materials with tailored properties have been produced for a wide range of applications, most notably the targeted delivery of drugs and other bioactive molecules [113], water remediation [114], analytical chemistry [115], and food packaging [116].

**Figure 5:**
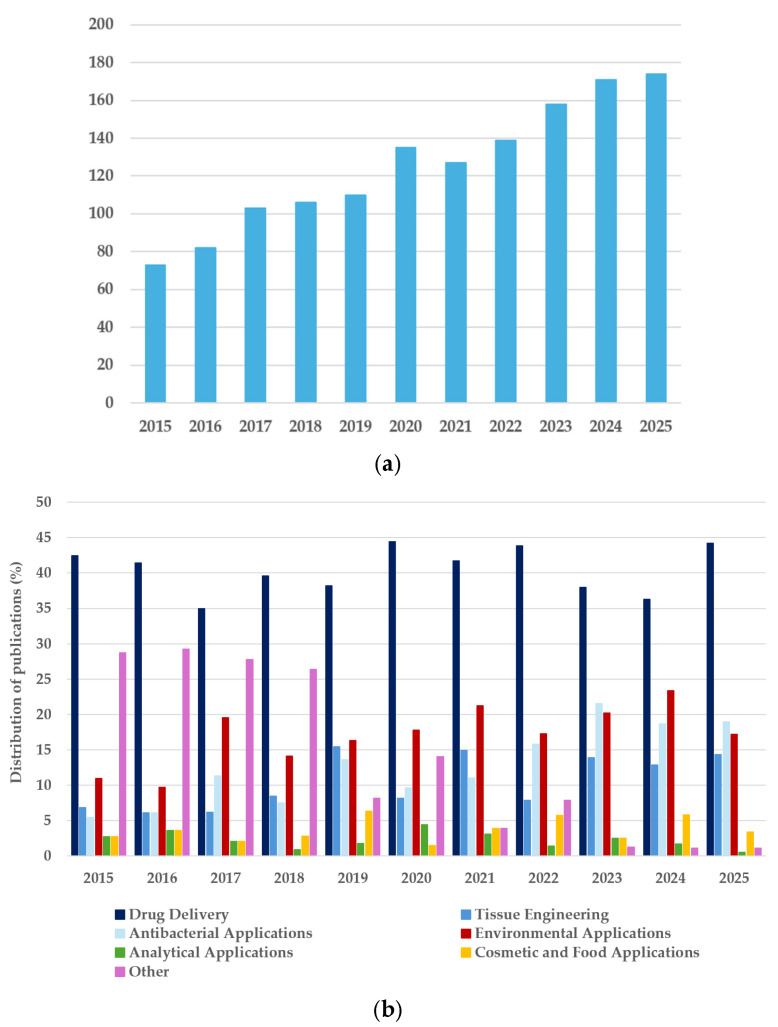
(**a**) Annual number of publications on cyclodextrin-grafted polysaccharides over the past decade, including articles, patents, and conference proceedings (source: Scopus database). The literature search was performed using the following keyword combination: cyclodextrin AND (polysaccharide OR chitosan OR alginate OR hyaluronic acid OR starch OR cellulose OR pectin OR dextran OR pullulan) AND (grafted OR conjugated OR functionalized OR immobilized OR cross-linked). (**b**) Percentage distribution of these publications across major research fields.

The growing interest **of** both academic and industrial communities is highlighted by the bibliometric data in Figure 5a. Over the past decade, the annual number of publications indexed in the Scopus database has shown a steady upward trend, confirming the critical role of CD-based polysaccharide systems in contemporary research. In particular, the percentage distribution reveals that the most extensively studied application areas are drug delivery and environmental science (Figure 5b).

Polysaccharides used in this context include chitosan, cellulose and its derivatives, starch, alginate, dextran, pullulan, pectin, and hyaluronic acid. Their conjugation with CDs has been extensively documented through various functionalization strategies. The most innovative approaches for each biopolymer are discussed below and summarized in Table 5, which details the grafting strategies, degrees of substitution, applications, and key advantages of these systems.

### 6.1. CDs Grafted onto Chitosan

**Figure 6:**
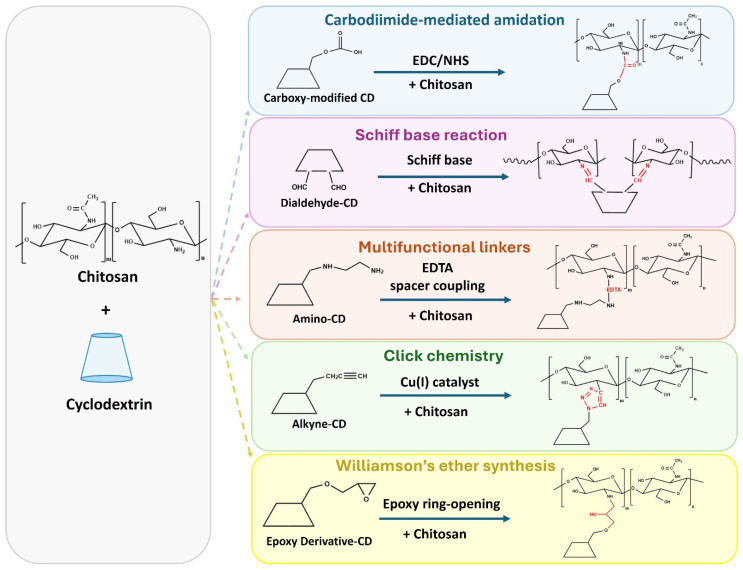
Examples of reactions for the grafting of cyclodextrins onto chitosan.

The most frequently used polysaccharide for grafting CDs is chitosan (CS), a linear copolymer produced by the deacetylation of chitin [165]. Chitin is primarily sourced from the exoskeletons of crustaceans and insects or the cell walls of fungi. CS is composed of D-glucosamine and N-acetyl-D-glucosamine units connected by β-(1–4) glycosidic bonds. The degree of deacetylation in commercial chitosan usually ranges from 70% to 95%. However, the poor solubility of chitosan in water and most common organic solvents results in low reactivity, limiting its practical applications. To enhance its solubility and expand its utility, chitosan is commonly modified through its amino and hydroxyl groups [166]. Among the most widely investigated derivatives are carboxymethylated [167], quaternized [168], acylated [169], and etherified chitosan. These modifications introduce hydrophilic or permanently charged functionalities, improving aqueous solubility across a broader pH range while preserving biocompatibility. Grafting CDs onto chitosan integrates the mucoadhesive, cationic, and bioactive nature of the polysaccharide with the host–guest inclusion capability of the CDs. This combination enables: (i) efficient encapsulation of hydrophobic molecules; (ii) enhanced drug delivery performance; (iii) broadened pollutant adsorption (targeting organic contaminants); (iv) improved physicochemical stability of the resulting hybrid materials. Traditional grafting has largely relied on nucleophilic substitution between monoactivated CD derivatives and the primary amino groups of chitosan. This typically yields randomly distributed pendant structures with limited architectural control. For example, Hou et al. prepared β-CD-grafted N-maleoyl chitosan via carbodiimide-mediated amidation. While not structurally precise, this remains a reliable standard for producing drug carriers [117]. Recently, the literature has shifted towards more versatile and precise synthetic strategies (Figure 6):

**Carbodiimide-mediated amidation (EDC/NHS)**: This involves activating the carboxyl groups on modified CDs (e.g., CM-β-CD) to react with chitosan’s amino groups. Yang et al. demonstrated that crosslink density in these systems governs swelling behavior and pH-responsive drug release [60]. Similarly, Xiao et al. used this method to create injectable pastes for localized postoperative tumor therapy [120]. Another variant involves the TEMPO-mediated oxidation of CDs to introduce carboxylic groups before coupling with chitosan, a method used to create hydrophobic cavities for toxin capture [118].

**Schiff base reaction (dialdehyde-CD)**: Gao et al. and Lou et al. reported that the use of sodium periodate to oxidize CDs into dialdehyde derivatives. These react with the amino groups of CS (or gallic acid-grafted CS) to form hydrogels via Schiff base linkages. This approach offers accelerated gelation and controlled release profiles [121,122].

**Multifunctional linkers (EDTA)**: Verma et al. synthesized a hierarchical adsorbent by tethering β-CD to chitosan through an EDTA spacer. This architecture enables the simultaneous removal of multiple pollutants: the EDTA moieties chelate heavy metals, while the CD cavities capture organic dyes [124].

**Click chemistry (CuAAC)**: Orthogonal click conjugation (Copper(I)-catalyzed Azide-Alkyne Cycloaddition) allows for highly regioselective attachment. Ez-Zoubi et al. demonstrated that this technique provides superior structural uniformity, controlled grating density, and fully preserved host cavities compared to traditional methods [123].

**Williamson’s ether synthesis**: Another promising approach involves activating β-CD with epichlorohydrin to form an epoxy derivative. The nucleophilic ring-opening of the epoxy group by the amino groups of chitosan establishes a robust covalent bond, significantly improving the encapsulation efficiency for hydrophobic drugs [119].

### 6.2. CDs Grafted onto Alginate

**Figure 7:**
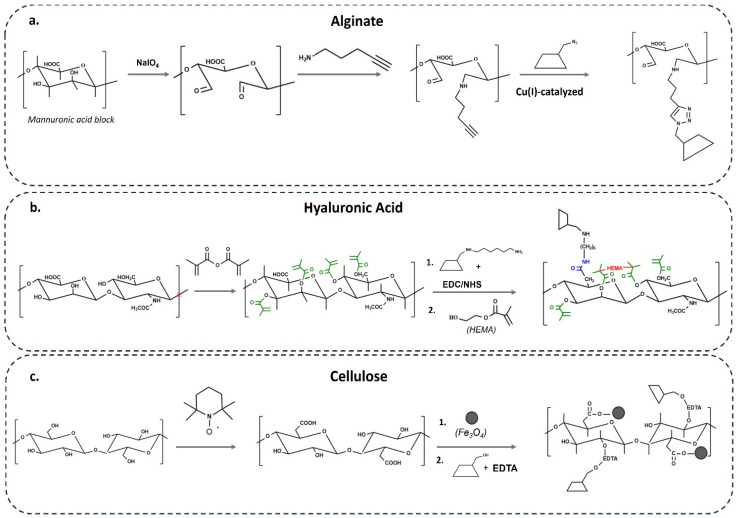
Examples of synthetic routes for cyclodextrin-modified polysaccharide systems. (**a**) CuAAC click chemistry strategy [16]: pre-oxidation of alginate with metaperiodate, functionalization with an alkyne linker, and subsequent grafting of N3-βCD via Cu(I)-catalyzed azide-alkyne cycloaddition. (**b**) Photocrosslinked networks: methacrylation of hyaluronic acid with methacrylic anhydride, conjugation via EDC/NHS with βCD-hexamethylenediamine, and radical crosslinking via copolymerization with HEMA monomer [135]. (**c**) Oxidation-mediated coupling: TEMPO-mediated oxidation of cellulose followed by simultaneous grafting of βCD (via EDTA-mediated ester bonds) and magnetite nanoparticles (via carboxylate-iron coordination interactions) [147].

Alginate (Alg) is an anionic polysaccharide produced by brown seaweed, consisting of a random sequence of two uronic acid blocks: α-L-guluronic acid (G), and the β-D-mannuronic acid (M), linked by 1,4-glycosidic bonds [170]. Alginate can form gels in the presence of divalent ions (such as Ca^2+^) through the formation of the so-called “egg-box” structures. These structures originate from the interaction between divalent ions and the carboxyl groups of the guluronate moieties. Due to its properties—including hygroscopicity, biocompatibility, biodegradability, and chelating ability—alginate is widely used in biotechnology, pharmaceuticals, textiles, and food industries [171,172,173]. However, pure alginate gels exhibit limitations regarding drug loading and release kinetics. CD-grafted alginates overcome these drawbacks by combining the gelling properties of the polysaccharide with the ability of CDs to form host-guest inclusion complexes with hydrophobic molecules. A prevalent method for coupling CD to Alg is via carbodiimide-mediated condensation. For instance, mono-6-deoxy-6-ethylenediamine-CD (CD-EDA) is frequently used, as its amino group reacts with carboxyl groups of Alg using the EDC/NHS activation system [129]. This approach was employed to create an Alg-β-CD host polymer (with a degree of substitution from 5 to 21%), which interacted with a polyacrylamide guest polymer functionalized with adamantane to obtain an injectable, self-healing supramolecular hydrogel [126]. Alternative synthetic routes include the Ugi-type multicomponent condensation, involving Alg, mono(6-amino-6-deoxy)-β-CD, formaldehyde, and cyclohexyl isocyanide in water at pH 3.6 [130]. Furthermore, Omtvedt et al. [16] developed a three-step strategy involving periodate oxidation of the polysaccharide, reductive amination with an alkyne-functionalized linker, and subsequent CuAAC click chemistry with azido-modified β-CD (Figure 7a). This method yielded well-defined pendant CD groups, while preserving the calcium-induced gelation capacity. More recently, Sogawa et al. reported a simplified one-step aqueous condensation where mono-6-amino-β-CD reacts directly with alginate carboxyl groups, offering a scalable route that maintains host-guest recognition [127]. The literature between 2016 and 2026 reflects a shift in interest from simple covalent “pendant” systems towards hybrid or supramolecular architectures where CDs modulate gelation, self-healing, and stimuli-responsiveness. Unlike chitosan-CD systems, the primary interest in Alg grafting lies in the resulting complex architecture. For example, Liu et al. demonstrated the entrapment of a valnemulin hydrochloride inclusion complex within an alginate nanogel matrix via ionic cross-linking, resulting in a pH-responsive delivery system [174]. Similarly, β-CD has been physically incorporated into sodium alginate matrices to improve the loading and thermal stability of enzymes like β-mannanase [131]. Advanced applications also involve polycarboxylic CD derivatives (esterified with citric acid or maleic anhydride) which interfere with Ca^2+^-mediated crosslinking to modulate the viscoelastic properties and morphology of the hydrogel [175]. Finally, dual-cross-linked systems have been developed where β-CD-grafted alginate forms reversible supramolecular crosslinks with hydrophobic moieties, complemented by a secondary ionic Ca^2+^ network. These materials exhibit self-healing and thermoresponsive behavior [128]. Similarly, dual pH/redox-responsive nanohydrogels utilize doxorubicin-conjugated alginate stabilized through CD inclusion complexation, demonstrating that CDs can actively participate in the supramolecular organization of the network rather than acting as passive pendants [176].

### 6.3. CDs Grafted onto Hyaluronic Acid

Hyaluronic acid (HA) is a long, unbranched polysaccharide composed of repeating disaccharide units of D-glucuronic acid and N-acetyl-D-glucosamine, with a molecular weight (MW) reaching up to 2 × 10^7^ Da. As a primary building block of the extracellular matrix, HA is involved in key biological processes such as cell signaling, wound repair, and tissue regeneration [177]. Its unique physicochemical profile, including biocompatibility, mucoadhesiveness, and viscoelasticity [178], makes it an ideal candidate for pharmaceutical applications. The release characteristics of HA-based systems can be finely tuned by modulating their degradation rates and mechanical properties [179]. Despite these advantages, the use of HA in environmental applications remains limited by high production costs, high-water solubility (which complicates recovery), and a low natural affinity for contaminants compared to biopolymers like alginate or chitosan. To overcome these limitations and enhance its controlled release capabilities, HA is frequently modified through CD conjugation. Several synthetic strategies have been employed for HA-CD conjugation:

**Carbodiimide chemistry (EDC/NHS)**: One of the most established strategies involves functionalizing β-CD at the C-6 position to introduce a terminal amino group (mono-6-deoxy-6-amino-β-CD). This amine then reacts with the activated carboxyl groups of HA via carbodiimide-mediated coupling. Bai et al. reported that this produces a stable HA-g-β-CD conjugate via amide bonds, preserving both the CD cavity and the HA backbone for host-guest interactions [133]. Similarly, Adaileh et al. utilized ethylenediamine-modified γCD (γCDEDA) to create self-assembled nanoparticles for curcumin delivery [134].

**Dual-Functionalized Systems**: Kaewruethai et al. expanded this approach by co-grafting both β-CD and PNIPAM chains onto the HA backbone. This resulted in a thermoresponsive nanogel where drug release is triggered by temperature-driven collapse of the PNIPAM segments [140].

**Photocrosslinked Networks**: Integrated CD units into covalent hydrogels by methacrylation of hyaluronic acid and subsequently performing radical copolymerization with methacrylate-functionalized CDs (Figure 7b). This combines the inclusion capability of CDs with the mechanical robustness of a photocrosslinked network [135].

**Carbamate Linkages**: Singh et al. reported an alternative route by activating β-CD with diphenyl carbonate and converting HA into its tetrabutylammonium salt (TBA) to enable dissolution in organic media. The resulting carbamate-linked conjugate preserves the native carboxylate groups of HA, which is crucial for CD44-mediated cell targeting [136].

**Polypseudorotoxane Scaffolds**: Hwang et al. developed a non-covalent approach where CD rings are treated on polymer chains associated with HA. These structures were then stabilized via the oxidative polymerization of dopamine, creating an injectable hydrogel with reinforced mechanical integrity [137].

Strategies for advanced click chemistry and green synthesis are also described in the literature.

**Click Chemistry (CuAAC)**: Kovačević et al. employed a regioselective click chemistry approach, targeting the C-6 hydroxymethyl groups of HA rather than the carboxyl functionalities. By propargylating HA and reacting it with azide-functionalized β-CD, they obtained a conjugate linked by 1,2,3-triazole bridges. This method ensures that the polyelectrolytic character of hyaluronic acid (the negative charge) remains fully intact [138].

**Aqueous Green Synthesis**: Recently, Bognanni et al. developed a sustainable synthesis performed entirely in aqueous media. Their HA-CD conjugates act as soluble nanocarriers that significantly enhance the cellular uptake of doxorubicin in CD44- overexpressing cells, demonstrating that the degree of substitution and HA molecular weight are critical factors for therapeutic efficacy [139].

### 6.4. CDs Grafted onto Cellulose

Cellulose is a renewable and highly abundant biopolymer derived from diverse biomass sources, including wood, tunicates, agricultural residues, and bacterial fermentation. Structurally, it is a linear homopolysaccharide of anhydro-D-glucose units linked through β-(1→4) glycosidic bonds, which promote extensive intra- and inter-molecular hydrogen bonding, resulting in a semi-crystalline and mechanically robust material [180]. Owing to its low toxicity, biocompatibility, and chemical versatility, cellulose is a premier sustainable platform for advanced functional materials in both biomedical (e.g., wound dressing, tissue engineering) and environmental fields (e.g., water purification, pollutant adsorption) [181,182,183] Grafting CDs onto cellulose yields multifunctional hybrid systems that synergistically combine the structural integrity of the cellulose matrix with the unique host–guest inclusion capabilities of CDs. This integration enables the selective capture of organic pollutants or the controlled delivery of poorly water-soluble drugs. Various strategies for the covalent grafting of cyclodextrins onto cellulose have been described in the literature:

**Polycarboxylic acids as crosslinkers**: A widespread, eco-friendly approach involves using polycarboxylic acids. Aguado et al. attached β-CD to α-cellulose through a two-step esterification process employing 1,2,3,4-butanetetracarboxylic acid (BTCA). The resulting covalently crosslinked fibers showed enhanced retention and a sustained release profile of guest molecules [141]. Similarly, Ghorpade et al. functionalized carboxymethylcellulose (CMC) with β-CD using citric acid as a green crosslinker, producing hydrogel films with superior drug release control compared to unmodified [145].

**Epichlorohydrin (EPI) linkage**: Epichlorohydrin remains a classic crosslinking agent. It has been used to graft β-CD onto cellulose beads under mild alkaline conditions, significantly increasing adsorption capacity towards Bisphenol A (BPA) through the formation of stable inclusion complexes [142]. A similar method was employed to create cellulose-g-β-CD/polypropylene glycol (PPG) supramolecular hydrogels, which exhibit remarkable temperature sensitivity and structural stability [146].

**Radical grafting and Fenton reactions**: Ares at al. described the modification of cellulose filter paper using β-CD functionalized with N-methylolacrylamide (NMA). By utilizing a Fenton reaction (Fe^2+^/H_2_O_2_) to generate surface radicals on the cellulose, they achieved covalent grafting of the CD-NMA monomers. The resulting material selectively adsorbs diclofenac, even in high-salinity environments [143].

**Oxidation-mediated coupling**: Introducing aldehyde or carboxyl groups via oxidation is an efficient route for CD grafting. Wang et al. developed a β-CD-functionalized composite using TEMPO-mediated oxidation, converting primary hydroxyl groups into reactive carboxyl functionalities for subsequent coupling (Figure 7c) [147]. Mota et al. used a similar TEMPO-oxidation strategy to incorporate β-CD and κ-carrageenan into multifunctional cellulose hydrogels [148].

**Click chemistry (CuAAC)**: Bagheri Renani and Zahedi reported the synthesis of β-CD-grafted cellulose nanofibers using Copper(I)-catalyzed Azide-Alkyne Cycloaddition (CuAAC). By introducing azide groups onto the cellulose and alkyne groups onto the CD, they created stable 1,2,3-triazole bridges, resulting in a highly efficient adsorbent for crystal violet removal [144].

Beyond covalent grafting, supramolecular strategies utilize physical interactions. Noe et al. developed composite hydrogels using cellulose nanocrystals (CNCs) as bio-based fillers. The CNC surfaces were modified with hydrophobic adamantane (Ad) groups, which then formed reversible host-guest inclusion complexes with β-CD units within a polymer matrix, acting as physical crosslinking points that reinforce the filler–matrix interface [149].

### 6.5. CDs Grafted onto Other Polysaccharides

In addition to chitosan, alginate, hyaluronic acid, and cellulose, CDs have been successfully anchored to other natural polymers, including starch, dextran, pullulan, and pectin. This enables the development of materials with customized physicochemical properties for biomedical and environmental applications.

#### 6.5.1. Starch

Starch is one of the most abundant renewable polysaccharides; it consists of linear amylose and branched amylopectin, whose structural organization determines properties such as crystallinity, swelling, and mechanical behavior [184]. Due to its high hydroxyl group density, biocompatibility, and ease of chemical modification, starch represents a sustainable platform for functional materials. However, native starch suffers from limitations such as poor water resistance and modest mechanical stability [185]. The integration of CDs into starch matrices combines the structural and film-forming properties of the polysaccharide with the host-guest inclusion capacity of CDs for hydrophobic molecules, generating fully bio-based systems of growing interest for biomedical, environmental, and packaging applications. CD-starch hybrid materials have been synthesized through various covalent and noncovalent strategies, leveraging the high density of hydroxyl groups and the structural compatibility of these two glucan-based polysaccharides. One of the most robust approaches involves the covalent immobilization of CDs on starch via chemical crosslinking. For example, Hao et al. prepared corn starch grafted with γ-CD via alkaline crosslinking reactions using epichlorohydrin as the crosslinking agent [151]. This agent facilitates the formation of stable ether bonds between the hydroxyl groups of both polymers, resulting in an insoluble network with a significantly higher adsorption capacity for organic dyes compared to native starch. Similarly, Zhang et al. reported adsorbent systems based on β-CD grafted on starch via covalent incorporation, using citric acid as a green crosslinking agent and sodium hypophosphite as the catalyst [152]. The presence of CD cavities provided additional host-guest binding sites beyond conventional surface adsorption, resulting in a significant improvement in the removal efficiency of dyes from aqueous solutions. Furthermore, starch-based adsorbents grafted with β-CD were synthesized through an analogous epoxide-based process using glycidyl methacrylate (GMA). Specifically, both the starch and the CDs were functionalized with GMA, and the resulting hydrogel was obtained by polymerizing acrylic acid salts to bridge the two components [154]. Unlike these chemically grafted systems, other studies have explored non-covalent or composite approaches in which CDs are incorporated into the starch structure without forming permanent bonds. Zheng et al. developed porous starch/β-CD composites by introducing CD molecules into the granular matrix, resulting in materials with increased specific surface area and modified physicochemical properties that improved adsorption performance [155]. More recently, Li et al. fabricated corn starch/β-CD composite nanoparticles through co-assembly processes, demonstrating that, even in the absence of covalent bonds, strong intermolecular interactions and structural confinement can stabilize the CD within the starch architecture [186]. These nanoparticles demonstrated efficient encapsulation of bioactive compounds, highlighting the potential of supramolecular organization for controlled-release applications.

#### 6.5.2. Dextran

Dextran is a natural, water-soluble polysaccharide primarily composed of α-(1→6)-linked D-glucose units with occasional α-(1→3) branches, typically synthesized by lactic acid bacteria from sucrose. Due to its high biocompatibility, low immunogenicity, and excellent aqueous solubility, dextran is widely used in biomedical applications, including plasma expanders, drug carriers, tissue engineering scaffolds, and injectable hydrogels [187]. Similar to other polysaccharides, the abundance of hydroxyl groups along its backbone facilitates versatile chemical modification, such as oxidation, esterification, etherification, and graft polymerization, enabling precise control over degradation rate and mechanical properties. Incorporating CDs into dextran matrices represents an effective strategy to enhance functionality by introducing molecular recognition capabilities. Consequently, dextran-CD hybrid systems combine the structural integrity and processability of the polysaccharide with the encapsulation properties of CDs. These advanced materials are utilized for controlled drug release, injectable depots and antimicrobial platforms. Various synthetic strategies have been developed, leveraging both covalent and supramolecular processes. One approach involves forming interpenetrating or cross-linked networks rather than direct grafting. Jalalvandi et al. developed degradable hydrogels by reacting oxidized dextran (dextran aldehyde) with amino-functionalized polymers containing CD units via Schiff base chemistry [156]. This yielded injectable matrices where dextran provides the structural framework, while CDs serve as inclusion sites for sustained drug release. A more direct integration was described by Slavkova et al., who prepared macroporous cryogels via free-radical copolymerization of dextran and acrylated β-CD under cryogenic conditions [157]. The covalent incorporation of the CD derivative generated stable porous scaffolds with significantly improved curcumin encapsulation efficiency. Similarly, D’Elia et al. designed injectable granular hydrogels by copolymerizing methacrylated dextran with methacrylated CD [158]. By exploiting the similarity between adamantine and CD, these materials have enabled affinity-controlled retention and sustained release of protein drugs for several weeks. An alternative strategy was reported by Shatan et al., who synthesized covalent β-CD-dextran conjugates through sequential functionalization steps. Dextran was tosylated and converted to Dex-EA via reacting with ethanolamine (EA), while β-CD was modified with vinyl sulfone before coupling [159]. The resulting hybrid coating for magnetic nanoparticles exhibited high aqueous stability and broad antimicrobial activity upon loading silver-sulfamethazine complexes. Finally, systems where CDs and dextran coexist without direct bonding have also been explored. Zhang et al. reported thermosensitive hydrogels containing oxidized dextran along with CD-drug inclusion complexes [160]. In this setup, CM-β-CD complexed ethinylestradiol, while oxidized dextran participated in the thermogelling network with chitosan and β-glycerophosphate (β-GP). These composites demonstrated improved solubilization of hydrophobic molecules through the cooperative action of both components.

#### 6.5.3. Pullulan

Pullulan is a natural, water-soluble, neutral polysaccharide produced by the fungus Aureobasidium pullulans. It consists of repeating maltotriose units linked by α-(1→6) glycosidic bonds, with internal α-(1→4) bonds within each trisaccharide block. This unique architecture confers high flexibility, excellent film-forming ability, and remarkable oxygen barrier properties, while maintaining biocompatibility and non-toxicity. Functionalizing pullulan with CDs is a highly effective strategy to expand its application range, particularly in targeted drug delivery, injectable hydrogels, and active packaging. Pullulan-CD hybrid materials are typically developed through covalent grafting, polymerization, or supramolecular assembly, leveraging the polysaccharide’s high hydroxyl group density and solubility. Direct chemical binding was achieved by Yang et al., who synthesized β-CD-grafted pullulan conjugates subsequently modified with glycyrrhetinic acid to yield amphiphilic polymers. These polymers self-assemble into liver-targeted nanoparticles [161]. In this architecture, the covalent attachment of β-CD provides stable host sites for drug inclusion, while the hydrophobic modification drives the formation of nanoparticles with prolonged circulation time and increased hepatic accumulation. A similar covalent approach was reported by Nonsuwan et al., where β-CD units were grafted onto pullulan and further functionalized with methacrylate groups to create photocrosslinkable derivatives [162]. Free-radical polymerization of these macromers produced hydrogels in which the CD cavities remained accessible, enhancing curcumic loading efficiency and ensuring its sustained release. Several studies have employed non-covalent strategies. Poudel et al. fabricated electrospun fibers from pullulan solutions containing HP-β-CD [188]. Here, the CD acts as a physically dispersed component that modifies the rheological behavior and fiber morphology, while facilitating active molecule encapsulation. Similarly, Hsiung et al. incorporated preformed CD-tetracycline inclusion complexes into pullulan nanofibers [189]. This approach exploits host-guest interaction to stabilize the antibiotic, relying on the pullulan matrix for rapid disintegration and release. More complex cooperative systems include pH-sensitive hydrogels based on pullulan networks, where β-CD mediates drug loading through supramolecular inclusion rather than permanent covalent anchoring [190].

#### 6.5.4. Pectin

Pectin is a naturally occurring anionic polysaccharide, primarily extracted from plant cell walls such as citrus peels and apple pulp. Its backbone consists of D-galacturonic acid units linked by α-(1→4) glycosidic bonds, which are partially methyl-esterified at the carboxyl groups. The complex architecture of pectin includes linear homogalacturonan regions and branched rhamnogalacturonan domains, which together dictate its physicochemical behavior. Due to its abundance of hydroxyl and carboxyl groups, pectin exhibits high hydrophilicity, mucoadhesiveness, and the ability to form gels in the presence of multivalent cations (e.g., Ca^2+^). These properties have led to widespread use in drug delivery, wound dressings, and environmental adsorbents [191]. Functionalizing pectin with CDs represents an effective strategy to enhance its interaction with hydrophobic molecules. The integration of pectin’s ionic gelling matrix with the molecular recognition of CDs enables the design of bio-derived hybrid materials with tunable adsorption and release kinetics. Direct chemical modification has been employed to create robust adsorbents. For example, Cesar Filho et al. synthesized pectin functionalized with β-CD through chemical coupling in DMF/sulfuric acid, subsequently forming a hybrid hydrogel with chitosan and polyvinyl alcohol [163]. In this system, the covalently tethered β-CD units act as host sites for aromatic compounds, while the polyelectrolyte complex formed with chitosan provides structural stability. Similarly, Chen et al. crosslinked β-CD to pectin using glutaraldehyde through aldehyde-mediated condensation [164]. This covalent linkage ensures that CD cavities remain accessible, significantly improving the adsorption of cholesterol and bile salts. Other studies focus on cooperative systems where CDs are incorporated without permanent covalent bonds. Nandal et al. developed pH-sensitive hydrogels composed of pectin and pullulan, where β-CD serves as a supramolecular host for phenothiazine derivatives [190]. Here, the polymer network regulates swelling and mechanical integrity, while the CD cavities control drug loading via inclusion complexation. Zheng et al. utilized a similar approach by encapsulating HP-β-CD/naringin inclusion complexes within pectin/alginate nanoparticles [192]. In this system, pectin serves as the biopolymer carrier, while CD enhances the solubility and stability of the hydrophobic flavonoid, resulting in improved antioxidant activity and cellular uptake.

The development of cyclodextrin-grafted polysaccharide systems is based on a close relationship between chemical design and final biomedical performance. A comparative analysis shows that, although all these platforms aim to combine the molecular inclusion capacity of cyclodextrins with the structural versatility of polysaccharides, their physicochemical behaviours and applications differ substantially depending on the synthetic routes and matrix topologies selected. The functionalisation of polysaccharides with CDs generally follows two distinct approaches: direct covalent grafting onto the polymer’s main chain, using bifunctional linkers, or the physical entanglement of CD-based polymers within a polysaccharide network. Direct grafting offers precise control over the density of CDs, but often requires the use of organic solvents or harsh reagents and conditions that may alter the polymer main chain to some extent. In contrast, mild and environmentally friendly preparation methods in an aqueous medium (for example, ionic gelation for alginate and chitosan) preserve the natural biocompatibility of the components, but produce semi-interpenetrating networks in which CD retention depends largely on steric entrapment, which may have a greater impact on the final properties of the system.

These synthetic choices dictate the network architecture, shifting the material properties between two extremes: on one hand, dense and organized hydrogels, and the other, open macromolecular networks. Specifically, when linear chains form highly organized or densely crosslinked networks stabilized by strong hydrogen bonds—as observed in certain cellulose derivatives, alginate, or chitosan matrices under specific conditions—the resulting structural compactness, with good mechanical profiles, restricts drug entrapment due to limited free volume and poor spatial accessibility of the cyclodextrin cavities. Concurrently, the dense network hinders the diffusion of water molecules, leading to a prolonged and controlled drug release governed by Fickian diffusion. On the other hand, a more amorphous and open network, typical of branched structures like modified starch or dextran, facilitates matrix swelling upon hydration. This enhanced swelling increases the spatial accessibility of the grafted cyclodextrins and creates larger interstitial spaces for non-specific drug entrapment. Consequently, the release profile shifts toward a non-Fickian (anomalous) transport mechanism, driven by the simultaneous contribution of polymer chain relaxation (swelling) and molecular diffusion.

Macromolecular charges further modulate the performance of these systems. Polyelectrolytes like chitosan are highly sensitive to the charge of the therapeutic agent and responsive to environmental stimuli such as pH and ionic strength. For instance, incorporating a counter-charged (negatively charged) drug enhances loading efficiency through the synergy of cyclodextrin inclusion and complementary ionic interactions, resulting in sustained release kinetics. Environmental changes in pH or ionic strength can screen these charges or alter polymer ionization, disrupting the electrostatic network and accelerating drug discharge. In contrast, incorporating a similarly charged (cationic) drug yields lower encapsulation efficiency due to electrostatic repulsion, leading to characteristically rapid release kinetics. Therefore, if high encapsulation and rapid release are required, the polysaccharide backbone must be branched and hydrophilic; if, on the other hand, the release is to be prolonged or targeted, the polymer must be linear or stimuli-responsive.

## 7. Applications of CD-Grafted Polysaccharides

**Figure 8:**
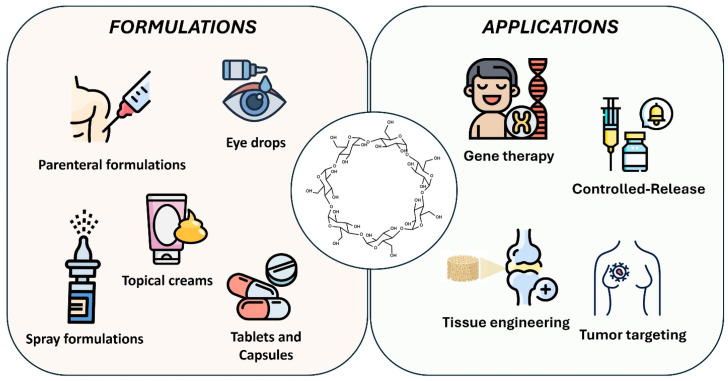
Examples of formulations and applications of CDs in the biomedical field.

Although CDs find applications across numerous industrial sectors, the most significant research-driven innovations over the past decade have emerged within the biomedical, pharmaceutical, and environmental fields. In the healthcare sector, CDs—both in their monomeric form and as sophisticated polymeric architectures—are utilized not only for the controlled delivery of small-molecule drugs and biopharmaceuticals (e.g., genes and peptides) but also for the engineering of stimuli-responsive systems for targeted therapies (Figure 8). Furthermore, they are integrated into scaffolds and biomaterials for tissue engineering and regenerative medicine, as well as in the treatment of complex pathologies, such as rare genetic disorders and lipid storage diseases [62,193].

The versatility and ease of functionalization of CDs allow for the design of innovative carriers that protect therapeutic agents from degradation while ensuring precise administration. On the environmental front, CDs and their polysaccharide-based derivatives represent an exceptionally promising strategy for the sequestration of organic and inorganic pollutants. These hybrid materials are characterized by high adsorption capacities, rapid binding kinetics, and the potential for multiple regeneration and reuse cycles. Consequently, they are increasingly applied in wastewater treatment and soil remediation, facilitating the efficient capture of heavy metals, pesticides, dyes, antibiotics, and emerging contaminants, often outperforming conventional adsorbent materials. Finally, owing to their inherent biodegradability and biocompatibility, CD-grafted polysaccharides are establishing themselves as benchmark materials for green chemistry and sustainable engineering [13,194].

In light of these advances, this review focuses on the recent synthetic strategies employed in the development of these hybrid materials and on their dual role in advanced medicine and wastewater treatment technologies.

### 7.1. Biomedical Applications

In the medical sector, hydrophobic compounds represent a significant hurdle for the effective administration and management of active pharmaceutical ingredients, especially regarding systemic delivery, cellular penetration, and controlled elimination (Figure 8). Numerous therapeutic agents—including polyphenols, natural derivatives, anticancer drugs, anti-inflammatories, analgesics, neuroprotectors, and lipophilic vitamins—exhibit poor bioavailability due to their limited solubility in biological fluids and rapid systemic clearance. It is estimated that approximately 40% of marketed drugs, and up to 90% of those in clinical development, are sparingly soluble in water. These compounds, classified under the Biopharmaceutical Classification System (BCS) as Class II (high permeability, low solubility) or Class IV (low permeability, low solubility), suffer from limited intestinal absorption, rapid metabolism, and low systemic availability, often coupled with suboptimal safety and tolerability profiles. This condition leads to several clinical challenges, such as the requirement for high dosages, undesirable side effects, increased treatment costs, and poor patient compliance [195,196]. By forming host-guest inclusion complexes, CDs can modulate the pharmacokinetic profiles of active ingredients, improving control over drug release rates, duration of action, and therapeutic efficacy. Therefore, their role in pharmacology is to enhance drug solubility and bioavailability, facilitating penetration through biological barriers and modulating pharmacodynamics [197]. Natural CDs (α-, β-, γ-) are generally considered safe and are listed as Generally Recognized as Safe (GRAS) substances by the FDA, as well as the European and US Pharmacopoeia. They are also recognized as food additives by the World Health Organization website [193]. Nevertheless, it is crucial to emphasize that not all CDs are suitable for all routes of administration. For example, native CDs—especially β-CD—can induce renal toxicity when administered parenterally and are therefore not recommended for this route [198]. In contrast, CDs are generally considered safe for oral administration due to their poor absorption in the gastrointestinal tract, resulting in minimal systemic toxicity. Parenteral administration, however, must be carefully evaluated: direct access to the bloodstream may lead to accumulation in the renal tubules and subsequent nephrotoxicity, especially with β-CD and its highly substituted derivatives (e.g., methyl-β-CD). High doses or prolonged treatment have been linked to renal damage and gastrointestinal disorders. In contrast, water-soluble derivatives such as HP-β-CD and SBE-β-CD have demonstrated a high safety profile, even at relatively high doses, and are commonly used in approved parenteral formulations for drugs such as diazepam, phenytoin, itraconazole, and diclofenac. Furthermore, increasing the bioavailability of guest molecules may elevate the risk of drug toxicity. While CDs can significantly enhance absorption, failure to properly adjust the dosage can lead to plasma concentrations that exceed the therapeutic window. Thus, the toxicological profiles of inclusion complexes must be rigorously evaluated, as the type of CD, its degree of substitution, and the nature of the complex play crucial roles in system safety. Currently, numerous products based on inclusion complexes with both native and functionalized CDs are FDA-approved and commercially available. Examples include Noxafil (an injectable solution using SBE-CD and posaconazole), Sporanox (utilizing HP-β-CD and itraconazole), Brexin (tablets containing β-CD and piroxicam), or Voltaren Oftabak eye drops (with HP-γ-CD and diclofenac sodium) [199]. Beyond standalone CDs, research has expanded into CD-containing composite systems with enhanced properties, such as higher encapsulation efficiency, stimuli-responsiveness, and site-specific action [200]. A particularly efficient class of materials is CD nanosponges. For example, Pivato et al. recently synthesized a nanosponge hydrogel by crosslinking β-CD with pyromellitic dianhydride. The anti-inflammatory drug piroxicam was loaded into this system, resulting in a sustained release governed by diffusion dynamics within the polymer network. This release is influenced by two main factors: the physical hindrance of the polymer network and the formation of inclusion complexes between β-CD and the drug. The authors highlight the synergistic benefits achieved through the crosslinking of CDs in such systems [201]. The synergistic properties of CDs and polysaccharides make these integrated systems ideal for a diverse range of pharmaceutical applications. For instance, Gao et al. developed hybrid hydrogels through the covalent attachment of CDs to gallic acid-functionalized CS, creating an innovative platform for the local delivery of CUR in treating bacterially infected wounds [121]. This system exhibited potent antimicrobial activity against Gram-positive and Gram-negative pathogens, including Staphylococcus aureus, Escherichia coli, and Pseudomonas aeruginosa. These results highlight the synergistic efficacy of the intrinsically antibacterial chitosan, antioxidant polyphenols, and CD-mediated complexation. Furthermore, in vivo experiments demonstrated accelerated wound healing, with significant tissue recovery within approximately 20 days due to the simultaneous elimination of pathogens and promotion of regeneration. The integration of CDs into polysaccharide matrices can also be achieved via polyelectrolyte complexes. Jiang et al. fabricated double-network hydrogels of cyclodextrin-grafted CS-AL through physical cross-linking [125]. This architecture exhibited a markedly improved swelling capacity, exceeding 800–1000% at physiological pH, while maintaining structural integrity. The presence of grafted β-CD increased the loading of hydrophobic drugs, achieving encapsulation efficiencies of about 85–90%. Drug release studies showed sustained profiles over several days, with cumulative release reaching 70–85% after 48–72 h and a reduced initial burst compared to non-CD systems. In the field of ophthalmic delivery, Deng et al. developed a copolymer hydrogel consisting of poly(2-hydroxyethyl methacrylate) (PHEMA) and methacrylated hyaluronic acid functionalized with β-CD (HA-β-CD) [135]. Designed as a therapeutic contact lens, this material exhibited high optical transparency (>90% transmittance), equilibrium water content of 60–75%, and excellent wettability. The incorporation of β-CD extended drug release to approximately one week, a significant improvement over conventional lenses, which typically deplete their payload within hours. Additionally, in vitro studies confirmed high cell viability (>90%), supporting its suitability for long-term ophthalmic use. Stimuli-responsive systems have also seen significant advancement. Reis et al. demonstrated that engineered cyclodextrin-starch hydrogels exhibit pronounced pH-responsive swelling, with higher ratios under mildly acidic conditions (pH ≈ 5–6) compared to physiological pH [154]. This enables controlled release tailored to inflamed or tumorous microenvironments. Similarly, Zhang et al. developed a thermosensitive injectable hydrogel composed of chitosan, oxidized dextran, and β-glycerophosphate (CS-ODex-β-GP) [160]. This system undergoes rapid in situ gelation at physiological temperature (36–37 °C) within 2–5 min. It successfully improved the aqueous solubility of ethinyl estradiol, providing sustained release for over 30 days with a minimal initial burst (about 20% in the first 24 h). A novel supramolecular approach was proposed by Nandal et al., using β-CD as a central mediator in pullulan/pectin hydrogels rather than as a covalently grafted moiety [190]. These hydrogels showed pH-dependent swelling, reaching 400–500% under intestinal conditions (pH ≈ 7.4) compared to <200% in gastric environments (pH 1.2). This allowed for targeted intestinal delivery of phenothiazine, with encapsulation efficiencies exceeding 80%. Similarly, Tahmasebi et al. developed magnetic sodium alginate/β-CD bio-nanocomposite microspheres for colon-targeted delivery [202]. The inclusion of magnetic nanoparticles provided structural stability and the potential for external guidance, while β-CD ensured high loading (80–90%) of hydrophobic anticancer agents. Finally, Quan et al. synthesized a semi-interpenetrating composite hydrogel based on carboxymethylcellulose, acrylamide, and β-CD [150]. This system exhibited exceptional water uptake, with equilibrium swelling reaching 1800–2200%. The β-CD moieties significantly increased the affinity for aromatic drug molecules, achieving loading capacities exceeding 200 mg g^−1^. The release followed a non-Fickian transport mechanism, where both diffusion and polymer relaxation governed the kinetics over 72 h.

### 7.2. Environmental Applications

Environmental pollution, driven by rapid industrialization and uncontrolled human activities, poses a critical threat to human health, biodiversity, and global ecosystems. Consequently, environmental remediation has become a top priority on major international political agendas [28,194,197]. Micropollutants—such as heavy metals, pesticides, dyes, persistent organic compounds, endocrine disruptors, and pharmaceutical residues—are toxic, bioaccumulative, and resistant to natural degradation, even at trace concentrations. These compounds enter water resources through industrial and urban effluents, accumulating in living organisms and exerting deleterious effects on the nervous, endocrine, reproductive, and cardiovascular systems. Research is currently focused on advanced, cost-effective solutions—including adsorption, membrane treatments, advanced oxidation processes, and hybrid approaches—for the simultaneous removal of organic and inorganic pollutants. Developing sustainable, accessible, and high-performance remediation technologies is essential to address this environmental emergency and safeguard natural resources [13,194]. Adsorption technologies have been widely adopted due to their efficiency and cost-effectiveness. In recent years, various practical adsorbents have been developed for wastewater treatment. In this context, CDs are particularly noteworthy due to their ability to form host-guest inclusion complexes with hydrophobic molecules—such as polycyclic aromatic hydrocarbons (PAHs), polychlorinated biphenyls (PCBs), and pesticides—as well as their capacity to interact with heavy metals. Crosslinked CDs are among the most advanced systems since they offer stable, water-insoluble three-dimensional structures with significantly higher adsorption capacities than native CDs. These structures enable the simultaneous removal of diverse contaminants. For example, a recent study utilized CD-based nanosponges (CD-NSs) synthesized using citric acid as a crosslinking agent and NaH_2_PO_4_ as a catalyst. These materials were evaluated as adsorbents for the dispersive solid-phase extraction (dSPE) of 48 organic pollutants—including PAHs, PCBs, organochlorine pesticides (OCPs), organophosphate esters (OPEs), antibacterial agents, and UV filters—from seawater and wastewater [203]. The study demonstrated the effectiveness of these nanosponges as sustainable sorbents for multi-contaminant extraction, showing high recovery rates across diverse chemical classes. This performance is attributed to the synergistic effect of the porous polymer network and the specific molecular recognition of the CD cavities. Consequently, CD-based nanosponges offer a sustainable, environmentally friendly, and robust solution for water remediation. Another study investigated the production of two CD-based nanosponges synthesized using different linkers: hexane-1,6-diamine (HD6) and dodecane-1,12-diamine (HD12). These were evaluated as adsorbents for the removal of the herbicide 2,4-dichlorophenoxyacetic acid (2,4-D) from aqueous solutions. Physicochemical characterization revealed that the longer-chain linker (HD12) resulted in a more compact nanosponge structure. Maximum adsorption for CD-HD6 and CD-HD12 was achieved at pH 4.0 and 3.0, respectively. Under these acidic conditions, the amino groups of the nanosponges are protonated (positively charged), while the 2,4-D molecules—depending on their pKa—interact with the matrix via electrostatic attraction. Although the adsorption isotherms differed, both materials exhibited a remarkable capacity for 2,4-D removal, with the HD12-based nanosponge demonstrating superior performance. Furthermore, an alkaline desorption procedure was developed, enabling complete regeneration and reuse of the adsorbents, making them promising candidates for the sustainable management of agricultural wastewater [204]. Growing attention has been directed towards the synthesis of cost-effective adsorbents capable of efficiently removing contaminants from both natural and wastewater. In this regard, polysaccharides have proven to be exceptionally promising materials. In particular, the adsorption of toxic compounds onto cyclodextrin-modified polysaccharides has been extensively exploited. Owing to their biodegradability, biocompatibility, and high chemical functionality, these systems are ideally suited for developing sustainable, eco-friendly, and highly selective adsorbents [114]. As previously discussed, the combination of CDs and polysaccharides yields various systems tailored to specific environmental requirements. For example, chitosan/β-CD beads have been synthesized for the adsorption of hexavalent chromium [Cr(VI)]. These β-CD-functionalized spheres demonstrated significantly higher removal efficiency compared to pristine chitosan beads, maintaining robust performance over four adsorption/desorption cycles. Therefore, they represent an effective class of adsorbents for the remediation of aqueous streams where Cr(VI) concentrations exceed regulatory limits [205]. Recently, Sogawa et al. reported a one-step aqueous synthesis of β-CD-substituted alginate via condensation between the carboxyl groups of alginate and amino-functionalized β-CD. This yielded a covalently grafted polysaccharide capable of robust host-guest recognition in water [127]. The modified alginate exhibited a degree of substitution in the range of 5–10% of the uronic acid units, which was sufficient to introduce a high density of inclusion sites while preserving solubility. Spectroscopic analyses confirmed the stable immobilization of the CD, and guest-binding studies demonstrated association constants (*K_a_*) between 10^3^ and 10^4^ M^−1^ for hydrophobic aromatic molecules, indicating an effective capture capacity. The material showed significantly improved adsorption compared to native alginate, with capacities reaching tens of milligrams per gram of polymer for model organic pollutants. Furthermore, as previously reported, Bagheri Renani and Zahedi developed β-CD-grafted cellulose nanofibers via Cu(I)-catalyzed azide-alkyne cycloaddition (click chemistry), producing a nanostructured adsorbent with permanently grafted host cavities [144]. These functionalized nanofibers exhibited superior crystal violet removal compared to unmodified cellulose, achieving adsorption capacities exceeding 250–300 mg g^−1^ and removal efficiencies above 95% under optimized conditions. Kinetic analysis indicated rapid adsorption, with equilibrium reached within 60–120 min, consistent with the high surface area and accessibility of the grafted CD sites. The adsorption process followed a Langmuir-type isotherm, suggesting monolayer binding dominated by host-guest inclusion and electrostatic interactions. Importantly, the material retained 80–90% of its original capacity over multiple regeneration cycles. Another innovation involved sodium alginate microspheres containing β-CD and multiwalled carbon nanotubes, used as a hybrid hydrogel adsorbent for the removal of perfluorinated compounds from water [132]. The composite showed high affinity for perfluorooctanoic acid (PFOA), with distribution coefficients (K_d_) in the range of 10^3^–10^4^ mL g^−1^, indicating strong partitioning even at trace concentrations (μg L^−1^ level). Unlike traditional powdered adsorbents, these millimeter-sized microspheres exhibited excellent mechanical stability and negligible mass loss, allowing for easy recovery. The presence of carbon nanotubes significantly accelerated mass transfer, reducing the time to achieve quasi-steady state to less than 120 min, while β-CD contributed to the selective inclusion of hydrophobic fluorinated chains. The versatility of CDs extends to starch-based systems used as fully bio-derived cationic flocculants for turbidity removal [153]. This material exhibited high charge density and strong bridging ability, achieving turbidity removal efficiencies of 95–99% at low dosages (5–20 mg L^−1^). Rapid clarification was observed within 5–10 min, significantly faster than untreated systems. The flocculant functioned effectively across a wide pH range (4–9), producing larger and denser flocs compared to native starch and resulting in residual turbidity values below 1–2 NTU, consistent with drinking water standards. Finally, the creation of complex systems based on multiple polysaccharides can yield more robust materials than single-polymer systems. Mota et al. developed an all-polysaccharide hydrogel composed of TEMPO-oxidized cellulose, κ-carrageenan, and β-CD [148]. The material exhibited extreme water absorption, with equilibrium swelling rates exceeding 3000–4000%, reflecting the highly porous and polyelectrolytic network. The incorporation of β-CD introduced hydrophobic sites, improving affinity for organic contaminants compared to cellulose-only hydrogels. Adsorption capacities reached 120–160 mg g^−1^, with equilibrium achieved within 180 min. Despite high hydration, the hydrogel maintained structural stability and could be reused, retaining 70–80% of its capacity over multiple cycles. These results highlight the potential of multi-polysaccharide CD hydrogels as sustainable, robust materials for water purification.

## 8. Conclusions and Future Perspectives

Cyclodestrins (CDs) and their derivatives have found widespread applications across a vast range of fields, including chemistry, pharmacy, medicine, food and beverage, cosmetics, chromatography, catalysis, biotechnology, agrochemistry, and environmental remediation. Today, they are integrated into numerous everyday products, from ibuprofen tablets and pertussis vaccines to smoking cessation aids and hair loss treatments. Furthermore, they serve as essential components in personal care products like toothpastes and shampoos, as well as in technical applications such as chromatographic columns, biopesticides, and bioadsorbents for water treatment. In line with recent scientific advances, CD-based systems—especially those integrating CDs with polysaccharide backbones—are becoming increasingly attractive for both biomedical and environmental applications. This review has provided a comprehensive overview of CDs, focusing on their unique structural characteristics and their ability to form host-guest inclusion complexes with various hydrophobic molecules. The diverse methodologies used for CD modification and their subsequent immobilization onto polysaccharide backbones have been thoroughly described. Because of the synergy between the host-guest inclusion capacity of CDs and the biocompatible, biodegradable, and versatile functional properties of polysaccharides enables the design of innovative hybrid architectures, these systems are highly adaptable, meeting diverse requirements across both clinical and ecological sectors.

Although laboratory results obtained thus far are highly encouraging, scaling up these systems to an industrial level requires significant optimization in terms of synthesis, purification, stability, and production costs, the latter of which currently remain prohibitive. Furthermore, it is crucial to rigorously evaluate the safety, biocompatibility, and long-term efficacy of these materials through extensive in vivo and in vitro studies, alongside validation testing on complex, real-world environmental matrices. A deeper understanding of the grafting mechanisms and supramolecular interactions governing CD-based polymeric networks is equally essential to optimize performance and expand their application range.

Looking ahead, academic and industrial interest in these systems is expected to grow steadily. The development of these hybrid platforms will likely accelerate through advanced synthesis and high resolution characterization techniques aimed at overcoming current limitations. In this scenario, CD-polysaccharide hybrids represent a promising frontier in the design of smart materials, offering innovative and sustainable solutions to increasingly urgent global challenges.

## Figures and Tables

**Table 2:** Thermal properties of α-, β-, and γ-CDs.

Cyclodextrin	Water Loss (°C)	Phase Transition (°C)	Degradation (°C)
α-CD [36]	74–121	134	270–320
β-CD [35,36]	87–110	222	250–330
γ-CD [37,38]	63–81	-	293–324

**Table 4:** Architectural designs, advantages, applications, and representative case studies of cyclodextrin-based systems.

Architecture	Advantages	Applications	References
**CD-based PolyRotaxanes (CD-PRs)**	High biocompatibility and structural versatility	Biomaterials and drug delivery systems	Li et al. [67]; Wankar et al. [90]; Ceccato et al. [91]; Higashi et al. [92]
	Increased in vitro diffusion and ex vivo skin permeability	Transdermal delivery of carvedilol	Cardoso et al. [93]
	Optimized structures to enhance the efficacy of the systems	Intracellular delivery of genetic/protein material (Cas9 ribonucleoprotein)	Taharabaru et al. [94]
**Cross-linked Cyclodextrins (C-CDs)**	Systems with excellent encapsulation capabilities and properties that are highly variable depending on the cross-linking agent	Sensing and analytical chemistry	Roy et al. [95]
	High porosity, robustness, and biocompatibility	Wastewater treatment (heavy metal removal)	Jia et al. [96]
	Ability to complex hydrophilic molecules; slow and controlled release; increased encapsulation efficiency of CDs	Drug delivery	Liu et al. [89]; Wehl et al. [97]; Pyrak et al. [98]; Dossi et al. [99]; Luppi et al. [100]; Haimhoffer et al. [101]
	Retain solubility in water/alcohols; insolubility facilitates recovery/recycling	Environmental remediation	Goyal et al. [102]; Dossi et al. [99]; Luppi et al. [100]
**CD-based Star Polymers (star-CDPs)**	Polymer arms can be engineered to respond to stimuli	Targeted drug delivery, gene delivery, diagnostic imaging, and environmental fields	Tian et al. [103]; Zhang et al. [104]
	Improves turbidity removal through polymer-particle interactions	Wastewater treatment	Rambabu Koyilapu et al. [105]
**CD-grafted Polymers (CD-GPs)**	Direct formation of inclusion complexes with drugs and bioactive compounds; combining the properties of CDs with those of polymers	Superior performance across numerous potential applications	Wankar et al. [90]; Liu et al. [106]
	Stimuli-responsive behavior	Medical applications	Wankar et al. [90]; Yan et al. [107]; Wang et al. [108]
	Improved heavy-metal removal efficiency	Environmental remediation	Zhang et al. [109]; Celebioglu et al. [110]

**Table 5:** Summary of polysaccharide types, grafted cyclodextrin derivatives, grafting strategies, degrees of substitution, fields of application, advantages and corresponding references.

Type of Polysaccharide	Cyclodextrin Derivative	Grafting Strategy	Degree of Substitution	Principal Applications	Advantages	References
Chitosan	ECH-β-CD, CM-β-CD, (6-OTs-βCD), Amino-CD, TEMPO-oxidized β-CD, dialdehyde-CD	EDC/NHS amidation, Schiff-base reaction, CuAAC click chemistry, epichlorohydrin coupling, EDTA linker	9.6–20.0% [117] 28–57% [118] 23.8% [119]	Drug delivery, injectable hydrogels, cancer therapy, wound healing, pollutant adsorption	Excellent biocompatibility, mucoadhesion, pH-responsive release, dual adsorption of organic pollutants and heavy metals	Yang et al. [60]; Hou et al. [117]; Xiao et al. [120]; Gomez-Maldonado et al. [118]; Gao et al. [121]; Lou et al. [122]; Ez-Zoubi et al. [123]; Verma et al. [124]; Wang et al. [119]; Jiang et al. [125]
Alginate	β-CD-EDA, β-CD-HDA, amino-β-CD, Maleic β-CD	EDC/NHS coupling, Ugi multicomponent reaction, CuAAC click chemistry, direct amidation	1.6–4.7% [16] 5–21% [126] 2.3% [127] 5.3% [128]	Injectable/self-healing hydrogels and nanogels for drug delivery, wound healing, enzyme immobilization, pollutant adsorption	Preserves Ca^2+^ gelation, self-healing properties, stimuli-responsive behavior	Omtvedt et al. [16]; Ren et al. [129]; Ren et al. [126]; He et al. [130]; Sogawa et al. [127]; Dhiman et al. [131]; Miao et al. [128]; Jiang et al. [125]; Zakaria et al. [132]
Hyaluronic acid	β-tosyl-CD, 6-amino-β-CD, γ-CD-EDA	EDC/NHS amidation, carbamate linkage, photocrosslinking, CuAAC, aqueous green synthesis	10% [133] 8.5% [134] 12.4% [135] 6.8% [136] 5.2% [137] 3.8% [138] 3.7–8.6% [139]	Targeted drug delivery, nanogels for wound healing, injectable hydrogels, supramolecular network	CD44 targeting, high biocompatibility, thermoresponsive systems	Bai et al. [133]; Adaileh et al. [134]; Kaewruethai et al. [140]; Deng et al. [135]; Singh et al. [136]; Kovačević et al. [138]; Hwang et al. [137]; Bognanni et al. [139]
Cellulose	ECH-β-CD, β-CD–BTCA, TOCN–βCD, TOC–βCD, Azide-β-CD, 6-acrylamido-β-CD, Acryloyl β-CD	BTCA esterification, citric acid crosslinking, epichlorohydrin coupling, TEMPO oxidation, CuAAC	89–171 μmol β-CD/g cellulose [141] 18.6% [142] 1.2–1.5 mmol β-CD/g cellulose [143] 11.1% [144]	Pollutant adsorption, smart hydrogels, drug delivery	High mechanical strength, renewable substrate, excellent adsorption capacity	Aguado et al. [141]; Vishwajeet et al. [145]; Lin et al. [142]; Wu et al. [146]; Ares et al. [143]; Wang et al. [147]; Mota et al. [148]; Bagheri Renani et al. [144]; May et al. [149]; Quan et al. [150]
Starch	ECH-β-CD, γ-CD, β-CD-GMA	Epichlorohydrin crosslinking, citric acid esterification, GMA grafting, supramolecular assembly	8.15% [151] 10.8% [152] 12.8% [153]	Pollutant adsorption, dye adsorption, encapsulation and drug release, food packaging	Renewable, inexpensive, fully bio-based	Hao et al. [151]; Zhang et al. [152]; Reis et al. [154]; Zheng et al. [155]; Salehin et al. [153]
Dextran	CM-β-CD, CD-functionalized polyaspartamide, methacrylated β-CD	Schiff-base chemistry, free-radical copolymerization, methacrylation, covalent conjugation	Not reported	Injectable hydrogels, protein/drug delivery, antimicrobial coatings for pathogen inhibition, water treatment	High aqueous solubility, injectable formulations, sustained release	Jalalvandi et al. [156]; Slavkova et al. [157]; D’Elia et al. [158]; Shatan et al. [159]; Zhang et al. [160]
Pullulan	βCD functionalized glycyrrhetinic acid, CM-β-CD, HP-β-CD	Covalent grafting, methacrylation, supramolecular assembly	8.6% [161]	Drug delivery, electrospun fibers, hydrogels for wound healing, PH-responsive system	Excellent film-forming ability, prolonged drug release, high biocompatibility	Yang et al. [161]; Nonsuwan et al. [162]
Pectin	GLU-β-CD, HP-β-CD	Chemical coupling, glutaraldehyde crosslinking, supramolecular incorporation	14% [163]	Hydrogels, cholesterol adsorption, drug delivery	Excellent gel-forming ability, pH-responsive systems	Filho et al. [163]; Chen et al. [164]

## Data Availability

No new data were created or analyzed in this study. Data sharing is not applicable to this article.
